# Long-Term Storage of Ti_3_C_2_T_x_ Aqueous Dispersion with Stable Electrochemical Properties

**DOI:** 10.3390/ma17225414

**Published:** 2024-11-06

**Authors:** Ting Peng, Ruiqing Wu, Bohai Wang, Tomasz Liskiewicz, Shengwei Shi

**Affiliations:** 1 Hubei Key Laboratory of Plasma Chemistry and Advanced Materials, School of Materials Science and Engineering, Wuhan Institute of Technology, Wuhan 430205, China; pt@xjie.edu.cn (T.P.); 22305010031@stu.wit.edu.cn (R.W.); 22205010032@stu.wit.edu.cn (B.W.); 2 School of Chemical and Environmental Engineering, Xinjiang Institute of Engineering, Urumqi 830002, China; 3 Faculty of Science and Engineering, Manchester Metropolitan University, Manchester M15 6BH, UK; 4 Key Laboratory of Optoelectronic Chemical Materials and Devices (Ministry of Education), Jianghan University, Wuhan 430056, China

**Keywords:** MXene, stability, long-term storage, sodium L-ascorbate, supercapacitor

## Abstract

MXenes possess high metallic conductivity and excellent dispersion quality and pseudocapcitance. Their good hydrophilicity makes them particularly suitable as eco-friendly inks for printing applications. However, MXenes are prone to oxidization in aqueous dispersions, and it is very important to improve their stability. Here, the long-term storage of MXene aqueous dispersions was realized by the introduction of sodium L-ascorbate (NaAsc) as the antioxidant. The preserved MXenes exhibited very stable electrochemical properties. Even after 60-day storage, the supercapacitor with preserved MXenes as the electrode still demonstrated an excellent specific capacitance of 381.1 F/g at a scan rate of 5 mV/s and a good retention rate of 92.6% after 10,000 consecutive cyclic voltammetry measurements, which was nearly the same as that of fresh MXenes. The results indicate a facile and efficient method to realize the long-term storage of MXene aqueous dispersions for mass use in future energy storage.

## 1. Introduction

MXene is a two-dimensional (2D) layered carbide/nitride, generally denoted by the chemical formula of M_n+1_X_n_T_x_, where M represents the early transition metal element, X stands for C/N, and T is the surface group (such as -OH, -F, -Cl, etc.) [1]. The diverse and tunable surface chemistry of MXene affords it valuable and distinctive properties [2,3]. For example, the etching of the MAX phase with HF or LiF/HCl results in an MXene with -F groups on the external surface [4,5], while the alkali treatment can be applied to prepare MXenes without -F groups [6]. In addition, surface functional groups can be regulated by annealing at high temperatures [7]. Due to their distinctive structure and excellent properties, MXenes have been broadly applied in different fields such as sensors [8,9,10,11,12], supercapacitors [13,14,15,16,17,18], metal-ion batteries [19,20,21,22], photo/electrocatalysis [23,24,25,26], electromagnetic interference shielding [27,28,29,30,31,32], optoelectronics [33,34,35,36,37], and others [38,39,40,41].

However, MXene is prone to degrade, especially in aqueous dispersions [42,43]. The key factor for the instability of MXene was considered to be oxidization in the presence of water, and the dissolved oxygen in water was found to be fatal to MXenes in aqueous dispersions [44]. A cost-effective and eco-friendly way for long-term storage of MXenes in aqueous solution was reported based on the hydration chemistry of nontoxic inorganic salts [42], in which the attacking of MXene by dissolved oxygen molecules was restricted by reducing the water activity, as the concentration of dissolved oxygen greatly decreased in a NaCl solution. To reduce the oxidization of MXenes, various methods have been applied. Preparing different MAX phases is a feasible way to improve the stability of MXene due to different structures and surface groups in obtained MXenes [45,46]. Refrigerated cryogenic storage also can effectively prolong MXene stability in water at high cost [47,48,49]. As the storage of MXenes in organic solvents can provide an environment with low oxygen content, the stability of MXenes can be obviously enhanced [50,51,52]. The passivation of the MXene surface via the deep eutectic solvents method can extend its lifetime to 28 weeks, which is effective not only for MXene in a solution but also for that in a dry state [53]. In addition, high temperature annealing can induce a structure change in MXenes, and remove the inherent water molecules; thus, the stability of the MXene aqueous dispersion is effectively prolonged [54,55]. Furthermore, since the oxidization starts from the edge defects, MXene edges have been generally modified by anionic groups such as polyphosphates, polyborates, or polysilicates, which allows MXenes to be stored for more than three weeks [56]. The storage of MXenes with citric acid can improve MXene stability up to 5 months, but a large amount of citric acid is required [57]. Although sodium L-ascorbate (NaAsc) has been reported to effectively preserve Ti_3_C_2_T_x_ MXenes for up to 21 days [58], there has not been enough investigation centered on a longer period of time, and the effect on the electrochemical property of MXenes has not been investigated.

Here, the long-term storage of MXene aqueous dispersions with NaAsc as the antioxidant was investigated mainly based on the consideration of electrochemical properties. The passivation of NaAsc to MXene nanosheet defects/edges protected the MXene from the reaction with dissolved oxygen in water. In addition, the strong interaction between NaAsc and water reduced the content of dissolved oxygen in the water. MXenes can be well stored for 60 days in the atmosphere without any degradation of their electrochemical properties. The specific capacitance still reaches 381.1 F/g at a scan rate of 5 mV/s after 60 days of storage with NaAsc, and the retention rate is 92.6%. The results indicated a low-cost, facile, and effective way to store MXene aqueous dispersions as well as great potential as eco-friendly inks for printing processes in the future. Thus, our findings propose an effective approach for the long-term storage of MXene with minimal attenuation of its electrochemical performance.

## 2. Experimental Section

### 2.1. Materials

Ti_3_AlC_2_ was purchased from Foshan Xinene Technology Co., Ltd. (Foshan, China). Lithium Fluoride (LiF, 99%) and Tetramethylammonium Hydroxide (TMAOH, 25%) were obtained from Shanghai Macklin Biochemical Technology Co., Ltd. (Shanghai, China). Hydrochloric acid (HCl, AR) was purchased from Sinopharm Chemical Reagent Co., Ltd. (Shanghai, China). Ethanol absolute (EtOH, AR) was obtained from Hubei Forton Science and Technology Co., Ltd. (Wuhan, China). NaAsc was bought from Shanghai Aladdin Reagent Co., Ltd. (Shanghai, China).

### 2.2. Preparation of MXenes

Take 3 g of LiF and Ti_3_AlC_2_ each, prepare 60 mL HCl (9 mol/L), add the prepared HCl to the three-necked flask, slowly add LiF to the three-necked flask, and stir with a constant-temperature magnetic stirrer for 10 min until LiF is completely dissolved in HCl; slowly add 3 g of Ti_3_AlC_2_ to the three-necked flask in the above step using an ice bath, and then place in an oil bath at 40 °C for continuous stirring for 48 h. After the reaction is completed, centrifuge the reactant at 10,000 rpm for 10 min, remove the supernatant, and add deionized water (DI water) to the centrifuge tube, centrifuge at speed for 10 min, and repeat the centrifugation 5–6 times until the pH of the supernatant is close to 6; remove the lower layer of precipitate and add 20 mL of TMAOH by hand to make it dispersed evenly, and stir the intercalation layer with a constant-temperature magnetic stirrer for 5 h. Divide the MXene aqueous dispersion after TMAOH intercalation into three centrifuge tubes, add EtOH and centrifuge twice at 10,000 rpm for 10 min each time, and then add DI water and centrifuge twice at 10,000 rpm for 10 min each time. After centrifugation, remove the supernatant, collect the lower precipitate, add DI water and shake it to make it evenly dispersed, ultrasonicate for 1 h, and finally centrifuge at 4000 rpm for 35 min. The dark green supernatant liquid is taken as a few-layer dispersion liquid (high-concentration Ti_3_C_2_T_x_ dispersion), and the product Ti_3_C_2_T_x_ powder can be obtained by vacuum filtration and drying of the dispersion liquid.

### 2.3. Preparation of MXene-NaAsc Aqueous Dispersion

To 50 mg of Ti_3_C_2_T_x_ powder, add 50 mL of DI water to ultrasonicate for 30 min, and prepare a uniformly dispersed 1 mg/mL Ti_3_C_2_T_x_ aqueous dispersion. Weigh 50 mg of NaAsc powder, add it to the Ti_3_C_2_T_x_ aqueous dispersion, and sonicate for 30 min to obtain a uniform 50 mL 1 mg/mL Ti_3_C_2_T_x_-NaAsc aqueous dispersion. To preserve the stability of the dispersion, the concentration of NaAsc must be consistently maintained at 1 mg/mL [58]. Then, transfer the NaAsc-MXene dispersion and the fresh MXene aqueous dispersion to transparent glass vials, and place them in a room temperature environment without any manual manipulation for future use.

Ti_3_C_2_T_x_ aqueous dispersion for different storage times was represented as Ti_3_C_2_T_x_-H_2_O-x or Ti_3_C_2_T_x_-NaAsc-x, in which x indicated the number of days.

### 2.4. Characterizations

Vacuum filter the freshly prepared Ti_3_C_2_T_x_ aqueous dispersion (Ti_3_C_2_T_x_-Fresh) and the Ti_3_C_2_T_x_ aqueous dispersion stored with NaAsc for 15 days (Ti_3_C_2_T_x_-NaAsc-15) and 30 days (Ti_3_C_2_T_x_-NaAsc-30) to obtain their powders for further characterizations. Conduct X-ray diffraction (XRD, Germany) to obtain the crystalline structure with a D8 ADVANCE X-ray diffractometer. Measure UV-vis spectra with Lambda 35 UV spectrophotometer Characterize the morphology using a JSM-5510LV scanning electron microscope (SEM, Tokyo, Japan) and JEM2100 transmission electron microscopy (TEM, Tokyo, Japan), and analyze the detailed elements by X-ray photoelectron spectroscopy (XPS, UK) with ESCALAB Xi+ X-ray photoelectron spectrometer.

### 2.5. Electrochemical Measurement

Preparation of electrodes: Grind 24 mg of Ti_3_C_2_T_x_ sample into powder, and then mix it with activated carbon and polytetrafluoroethylene (PTFE) in a mass ratio of 8:1:1; add a drop of N-methylpyrrolidone (NMP) to make it fully ground, and spread it evenly on 1 × 1.5 cm^2^ foam copper substrate, under the pressure of 10 MPa. Press the weighed working electrode on a tablet press for 1 min, and then put it into a vacuum drying oven to dry to constant weight. Soak the prepared electrode in 1 mol/L H_2_SO_4_ electrolyte for 12 h to ensure that the electrolyte can fully penetrate the electrode material.

Electrochemical measurements: All electrochemical measurements were performed by a CHI660E electrochemical workstation (Shanghai Chenhua Instrument Co., Ltd., Shanghai, China). Three-electrode configuration was applied for electrochemical measurements, with Ti_3_C_2_T_x_ as the working electrode, Ag/AgCl as the reference electrode, activated carbon as the counter electrode, and 1 mol/L H_2_SO_4_ as the electrolyte. Cyclic voltammetry (CV) at different scan rates of 5–200 mV/s, galvanostatic charge/discharge (GCD) at 1–5 A/g, and CV cycling tests at 50 mV/s scan rate were measured. The volumetric capacitance is determined from the CV data by using the following equation:

(1) $$C=\frac{1}{\Delta V}\int \frac{\mathrm{idV}}{\nu }$$

where C is the normalized capacitance, i is the current density, ν is the voltage scan rate, V is the voltage, and ΔV is the voltage window.

## 3. Results and Discussion

**Figure 1:**
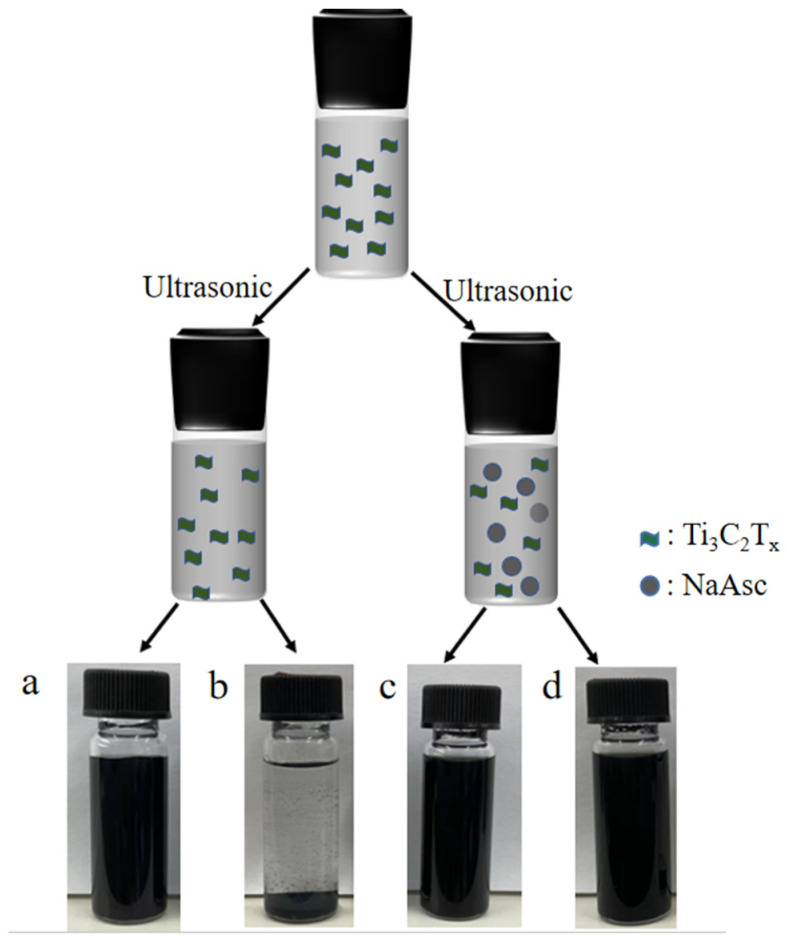
Photographs of Ti_3_C_2_T_x_ aqueous dispersions by ultrasonic treatment. (**a**) Ti_3_C_2_T_x_-H_2_O-0; (**b**) Ti_3_C_2_T_x_-H_2_O-30; (**c**) Ti_3_C_2_T_x_-NaAsc-0; (**d**) Ti_3_C_2_T_x_-NaAsc-30.

Figure 1 shows the preparation of the Ti_3_C_2_T_x_ aqueous dispersion by ultrasonic treatment. The fresh Ti_3_C_2_T_x_ aqueous dispersion was black whether NaAsc was added or not. Ti_3_C_2_T_x_-H_2_O showed poor stability due to the self-stacking of the MXene in the aqueous dispersion. After 30-day storage in the atmosphere, a large amount of precipitations were found in Ti_3_C_2_T_x_-H_2_O-30, resulting in the color change to faint black, while there was no precipitation in Ti_3_C_2_T_x_-NaAsc-30, and it remained a black and uniform dispersion after 30 days, indicating the improved stability of Ti_3_C_2_T_x_, which may result from the restricted self-stacking of Ti_3_C_2_T_x_ with the introduction of NaAsc.

Ascorbate anions preferred to combine with the edges or defects of Ti_3_C_2_T_x_ by electrostatic interaction, which thus protected Ti_3_C_2_T_x_ from the oxidization. In addition, the dissolved oxygen was considered to be responsible for the poor stability of Ti_3_C_2_T_x_ in aqueous dispersions [44]. As the hydration between Na ions and water prevailed in the competition with that between oxygen and water, there was less dissolved oxygen in Ti_3_C_2_T_x_-NaAsc than in Ti_3_C_2_T_x_-H_2_O [42]. Therefore, Ti_3_C_2_T_x_-NaAsc-30 demonstrated much better stability than Ti_3_C_2_T_x_-H_2_O-30. TEM images of Ti_3_C_2_T_x_-Fresh and Ti_3_C_2_T_x_-H_2_O-30 were also given in Figure S1. Smooth edges and distinct layered structures can be found in Ti_3_C_2_T_x_-Fresh, while they began to blur and even were not observed in Ti_3_C_2_T_x_-H_2_O-30, and many small fragments appeared due to oxidization.

**Figure 2:**
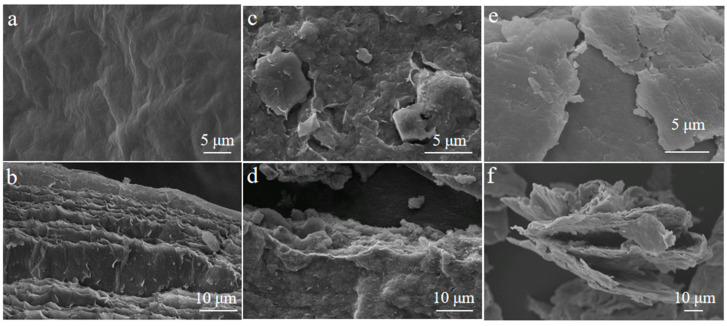
SEM images. (**a**) Ti_3_C_2_T_x_-Fresh (surface); (**b**) Ti_3_C_2_T_x_-Fresh (cross-section); (**c**) Ti_3_C_2_T_x_-H_2_O-30 (surface); (**d**) Ti_3_C_2_T_x_-H_2_O-30 (cross-section); (**e**) Ti_3_C_2_T_x_-NaAsc-30 (surface); (**f**) Ti_3_C_2_T_x_-NaAsc-30 (cross-section).

To have a better understanding of the morphology change between and after oxidization, SEM images were given for Ti_3_C_2_T_x_-Fresh, Ti_3_C_2_T_x_-H_2_O-30, and Ti_3_C_2_T_x_-NaAsc-30 in Figure 2**.** The original Ti_3_C_2_T_x_-Fresh showed a complete large-size layered structure with a very smooth surface and some clear folds (Figure 2a,b). However, Ti_3_C_2_T_x_-H_2_O-30 exhibited a fragmented small-size structure with a lot of small white matter on the surface, which may result from TiO_2_ formed at the edge of Ti_3_C_2_T_x_ (Figure 2c,d) [58]. Ti_3_C_2_T_x_-NaAsc-30 still exhibited a large-size layered structure with a smooth surface, and the layered structure was loose; moreover, there was no white matter as was the case in Ti_3_C_2_T_x_-H_2_O-30, which meant that the original structure of fresh Ti_3_C_2_T_x_ was kept well in Ti_3_C_2_T_x_-NaAsc-30 (Figure 2e,f).

**Figure 3:**
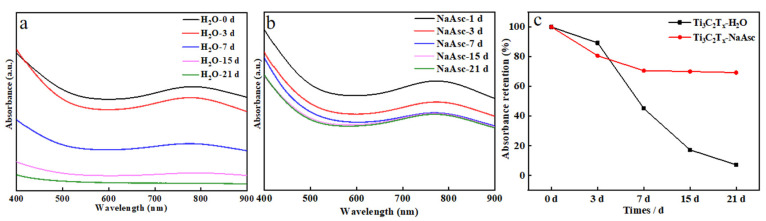
UV−vis spectra of MXene with different storage conditions. (**a**) Ti_3_C_2_T_x_-H_2_O; (**b**) Ti_3_C_2_T_x_-NaAsc; (**c**) change of relative absorption intensity at 773 nm with storage time for Ti_3_C_2_T_x_-H_2_O and Ti_3_C_2_T_x_-NaAsc.

Furthermore, the absorption spectra for both Ti_3_C_2_T_x_-H_2_O and Ti_3_C_2_T_x_-NaAsc were investigated as shown in Figure 3, and the evolution of the characteristic absorption of Ti_3_C_2_T_x_ at 773 nm was thus compared [59]. For Ti_3_C_2_T_x_-H_2_O, the absorption intensity decreased very fast with the storage time, and it was less than half of the original intensity after 7 days, and there was nearly no absorption feature after 21 days, while Ti_3_C_2_T_x_-NaAsc exhibited a very stable absorption intensity with the storage time, the absorption intensity gradually became stable after 3 days, and it still kept 75% of the initial intensity even after 21 days. The introduction of NaAsc obviously delayed the reduction of the absorption intensity with the storage time for Ti_3_C_2_T_x_-NaAsc.

**Figure 4:**
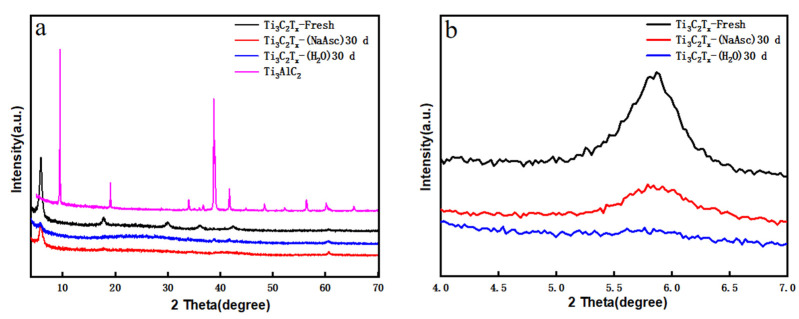
XRD patterns for Ti_3_C_2_T_x_ and Ti_3_AlC_2_ in different storage conditions. (**a**) Full angles and (**b**) low angles.

Figure 4 shows the comparison of XRD patterns for Ti_3_AlC_2_, Ti_3_C_2_T_x_-Fresh, Ti_3_C_2_T_x_-H_2_O-30, and Ti_3_C_2_T_x_-NaAsc-30. The (002) peak shifted from a high value in Ti_3_AlC_2_ to a low value in Ti_3_C_2_T_x_-Fresh, and the typical peak around 40° for Al element disappeared in Ti_3_C_2_T_x_-Fresh, indicating the successful preparation of Ti_3_C_2_T_x_ with high quality. For Ti_3_C_2_T_x_-NaAsc-30, the (002) peak showed a slightly lower angle than Ti_3_C_2_T_x_-Fresh; in addition, its intensity decreased a little bit, resulting from the oxidization of Ti_3_C_2_T_x_ to a certain extent after 30-day storage with NaAsc, and the loose crystal structure was confirmed by SEM in Figure 2f. As a comparison, for Ti_3_C_2_T_x_-H_2_O-30, its (002) peak totally disappeared as the layered structure was destroyed during storage, which was consistent with the SEM images in Figure 2c,d.

According to the Bragg equation

(2) 2dsinθ=nλ

where d is the crystal plane spacing, θ is the angle between the incident X-ray and the corresponding crystal plane, λ is the wavelength of the X-ray, and n is the diffraction series, Ti_3_C_2_T_x_-Fresh and Ti_3_C_2_T_x_-NaAsc-30 both showed the (002) peak at 5.86°, and the interlayer spacing of Ti_3_C_2_T_x_ nanosheets can be calculated to be 1.52 nm before and after the storage from Equation (2). Combining the SEM and XRD results, the stacking of Ti_3_C_2_T_x_ nanosheets can be well suppressed with the introduction of NaAsc, which is beneficial to ion transport in electrochemical applications.

**Figure 5:**
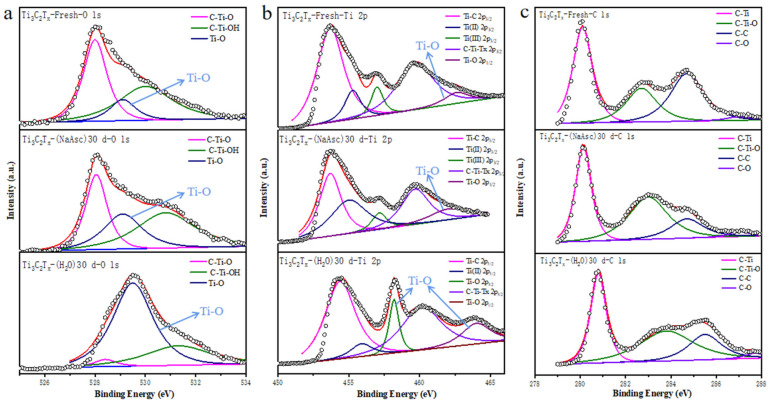
XPS of Ti_3_C_2_T_x_-Fresh, Ti_3_C_2_T_x_-H_2_O-30, and Ti_3_C_2_T_x_-NaAsc-30. (**a**) O 1s, (**b**) Ti 2p, (**c**) C 1s.

XPS measurement was conducted on Ti_3_C_2_T_x_-Fresh, Ti_3_C_2_T_x_-H_2_O-30, and Ti_3_C_2_T_x_-NaAsc-30 to understand what happened to Ti_3_C_2_T_x_ in the aqueous dispersion with the storage time (Figure 5). O 1s features were attributed to C-Ti-O (528.0 eV), C-Ti-OH (530.0 eV), and Ti-O (529.2 eV) for Ti_3_C_2_T_x_-Fresh (Figure 5a). The existence of Ti-O partially resulted from the binding of Ti to surface functional groups (-OH or -O-) of Ti_3_C_2_T_x_, and also from the oxidization during the preparation of Ti_3_C_2_T_x_. Ti-O accounted for 15.1% of the oxygen content in Ti_3_C_2_T_x_-Fresh, while it obviously increased to 70.2% in Ti_3_C_2_T_x_-H_2_O-30, indicating severe oxidization in a pure aqueous dispersion. The Ti-O content was found to be 31.2% in Ti_3_C_2_T_x_-NaAsc-30, indicating that the introduction of NaAsc effectively suppressed the oxidization of the MXene. Furthermore, Ti 2p features were divided into Ti-C (453.7 eV), Ti(II) 2p_3/2_ (455.3 eV), Ti(III) 2p_3/2_ (457.0 eV), and Ti-O 2p_1/2_ (463.5 eV) (Figure 5b). Ti-O binding in Ti 2p_1/2_ increased significantly in Ti_3_C_2_T_x_-H_2_O-30, and Ti(III) 2p_3/2_ basically shifted to the Ti-O 2p_3/2_ binding. In Ti_3_C_2_T_x_-NaAsc-30, Ti-O 2p_1/2_ showed quite similar features with those of Ti_3_C_2_T_x_-Fresh, which was consistent with the results in O 1s. C 1s showed nearly the same features as Ti_3_C_2_T_x_-NaAsc-30, Ti_3_C_2_T_x_-Fresh, and Ti_3_C_2_T_x_-H_2_O-30 (Figure 5c).

In addition, the conductivity of Ti_3_C_2_T_x_ was measured, which is an important parameter for MXenes, and Table S1 listed the conductivity for Ti_3_C_2_T_x_-Fresh, Ti_3_C_2_T_x_-H_2_O, and Ti_3_C_2_T_x_-NaAsc with the storage time. It was found that the conductivity of Ti_3_C_2_T_x_-NaAsc stayed at 87.5% in Ti_3_C_2_T_x_-Fresh after 30 days, while it decreased to less than 10^−3^ S m^−1^ in Ti_3_C_2_T_x_-H_2_O-15. The significant decline in conductivity can also be utilized as a preliminary predictive method for evaluating variations in the stability of MXene dispersions [60].

**Figure 6:**
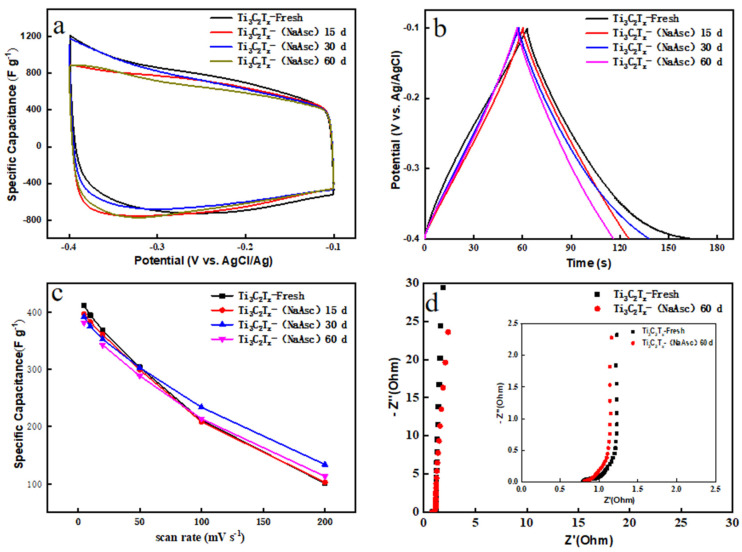
Electrochemical measurements for Ti_3_C_2_T_x_. (**a**) CV curves at 5 mV/s; (**b**) GCD curves at 1 A g^−1^; (**c**) specific capacitance with the scanning rate; (**d**) EIS curves.

The stable MXene aqueous dispersion demonstrated potential for the long-term storage of MXenes; moreover, it was quite suitable for the printing process as eco-friendly conductive ink. To understand how the preserved Ti_3_C_2_T_x_ still worked for high-performance supercapacitors, electrochemical measurements were conducted for Ti_3_C_2_T_x_ with different storage conditions. Figure S2 shows the CV curves for Ti_3_C_2_T_x_-Fresh, Ti_3_C_2_T_x_-NaAsc-15, Ti_3_C_2_T_x_-NaAsc-30, and Ti_3_C_2_T_x_-NaAsc-60 under different scanning rates, from which all CV curves showed a similar shape with the typical feature of an electric double-layer capacitor (EDLC), and Figure 6a shows the comparison of CV curves for all samples at 5 mV/s.

Table 1 lists the values of specific capacitance for the different samples. For Ti_3_C_2_T_x_-Fresh as the electrode, the specific capacitance was 411.4 F/g, and it was 396.3, 391.3, and 381.1 F/g for Ti_3_C_2_T_x_-NaAsc-15, Ti_3_C_2_T_x_-NaAsc-30, and Ti_3_C_2_T_x_-NaAsc-60, respectively. Compared with the fresh Ti_3_C_2_T_x_, the specific capacitance kept 96.3%, 95.1%, and 92.6% of the original value after the storage of 15, 30, and 60 days, respectively. Even at a high scanning rate of 200 mV/s, Ti_3_C_2_T_x_-NaAsc still showed a large specific capacitance. For example, after 60-day storage, Ti_3_C_2_T_x_-NaAsc-60 demonstrated 112.8 F/g, which was even higher than Ti_3_C_2_T_x_-Fresh at the same scanning rate. Figure S3 shows the GCD curves for Ti_3_C_2_T_x_-Fresh, Ti_3_C_2_T_x_-NaAsc-15, Ti_3_C_2_T_x_-NaAsc-30, and Ti_3_C_2_T_x_-NaAsc-60 at different current densities. They all demonstrated similar and symmetric triangular curves, indicating good capacitance characteristics and reversibility, and the preserved Ti_3_C_2_T_x_ exhibited almost the same GCD behavior as the fresh Ti_3_C_2_T_x_ (Figure 6b). Furthermore, the cyclic stability was characterized at 50 mV/s (Figure S4), and after 10,000 CV cycles, the specific capacitance was maintained at 99.4%, 99.6%, 99.0%, and 99.5% for Ti_3_C_2_T_x_-Fresh, Ti_3_C_2_T_x_-NaAsc-15, Ti_3_C_2_T_x_-NaAsc-30, and Ti_3_C_2_T_x_-NaAsc-60, respectively. Figure 6c shows the evolution of specific capacitance with the scanning rate. A slightly reduced specific capacitance was observed for Ti_3_C_2_T_x_ after the storage with NaAsc, which may be due to the unsaturation of NaAsc in the aqueous solution. Therefore, the defects of Ti_3_C_2_T_x_ were not totally combined with ascorbate anions. Related studies indicated that the best antioxidant effect on MXenes is obtained when the salt solution reaches saturation [42]. Figure 6d shows the comparison of EIS curves for Ti_3_C_2_T_x_-Fresh and Ti_3_C_2_T_x_-NaAsc-60, which exhibit quite similar EIS curves, indicating that Ti_3_C_2_T_x_ can be kept well in an aqueous dispersion long term with the introduction of NaAsc. Figure S5 and Table S2 show the comparison of electrochemical properties for Ti_3_C_2_T_x_-fresh and Ti_3_C_2_T_x_-H_2_O-30, indicating bad performance without the introduction of NaAsc.

The effect of the antioxidant is to protect the edges and defects of Ti_3_C_2_T_x_ from water and oxygen [58]. When an antioxidant was used, such as an anionic surfactant SDS [61], Tris-HCl [62], or NaAsc [58], they preferred to interact with positively charged groups on the Ti_3_C_2_T_x_ surface, which obstructed the interaction between water/oxygen molecules and Ti/C constituents within the Ti_3_C_2_T_x_; thus, the stability of Ti_3_C_2_T_x_ was greatly improved, and its electrochemical performance can be well kept. Here, the NaAsc-treated dispersion maintained colloidal stability even after 60 days at room temperature, with the 2D layered structure preserved as evidenced by SEM, and the stored Ti_3_C_2_T_x_ exhibited the specific capacitance of 381.1 F/g after 10,000 cycles with a capacitance retention of 92.6%, which was comparable to that of the fresh Ti_3_C_2_T_x_. Although Ti_3_C_2_T_x_ treated with hyperbranched polyethylene ionomers can be stably preserved in both aqueous and organic phases for a duration of 100 days, the data are absent regarding the electrochemical performance of long-term storage [63]. In addition, the functionalization of Ti_3_C_2_T_x_, such as with an amino-functionalized MXene, is a feasible way to improve its electrochemical performance through surface modification. Moreover, the stability of Ti_3_C_2_T_x_ can be enhanced as well [64]. Table 2 compares the electrochemical performance of the Ti_3_C_2_T_x_ aqueous dispersion with different antioxidant methods.

## 4. Conclusions

In summary, we investigated the effect of NaAsc on the long-term storage of Ti_3_C_2_T_x_, emphasizing its electrochemical performance across various storage stages. The findings demonstrated that NaAsc significantly enhanced the stability of Ti_3_C_2_T_x_ in aqueous dispersions, with ascorbic ions interacting with the cationic groups on the MXene surface, effectively protecting it from water and oxygen. Remarkably, even after 60 days of storage, its layered structure, dispersibility, and electrochemical properties remained largely intact. To assess electrochemical performance, the specific capacitance of supercapacitors utilizing MXenes as electrodes reached 381.1 F/g at a scan rate of 5 mV/s, with a capacitance retention rate of 92.6% after 10,000 CV cycles, comparable to that of fresh MXenes. Thus, our findings propose an effective approach for the long-term storage of MXenes with minimal attenuation of their electrochemical performance, which shows great potential for large-scale application of MXenes in energy storage.

## Figures and Tables

**Table 1:** Specific capacitance (F/g) of Ti_3_C_2_T_x_ at 1M H_2_SO_4_ electrolyte.

	5 mV/s	10 mV/s	20 mV/s	50 mV/s	100 mV/s	200 mV/s
**Ti_3_C_2_T_x_-Fresh**	411.4	394.2	368.5	303.5	211.1	100.7
**Ti_3_C_2_T_x_-NaAsc-15**	396.3	382.7	360.5	299.2	208.2	102.5
**Ti_3_C_2_T_x_-NaAsc-30**	391.3	374.7	352.4	302.6	233.4	133.6
**Ti_3_C_2_T_x_-NaAsc-60**	381.1	364.7	342.5	288.5	213.5	112.8

**Table 2:** Electrochemical performance of Ti_3_C_2_T_x_ with different antioxidant methods.

Antioxidant	Time (Day)	Electrolyte	Rate (mV/s)	Capacitance (F/g)	Retention	Ref
**SDS**	35	3 M H_2_SO_4_	-	~300 ^a^	95.4%	[61]
**Tris-HCl**	35	3 M H_2_SO_4_	-	251.6 ^a^	94.6%	[62]
**HPI**	-	[EMIM]^+^ [BF_4_]^−^	2	220 ^b^	-	[63]
**NaAsc**	60	1 M H_2_SO_4_	5	381.1 ^b^	92.6%	Here

All experimental conditions were maintained at room temperature, with a and b representing the calculations of specific capacitance derived from GCD and CV methods, respectively.

## Data Availability

The original contributions presented in the study are included in the article/Supplementary Materials, further inquiries can be directed to the corresponding authors.

## Supplementary Materials

The following supporting information can be downloaded at: https://www.mdpi.com/article/10.3390/ma17225414/s1, Figure S1: TEM images. (a) Ti_3_C_2_T_x_-Fresh; (b) Ti_3_C_2_T_x_-H_2_O-30. Figure S2: CV curves. (a) Ti_3_C_2_T_x_-Fresh; (b) Ti_3_C_2_T_x_-NaAsc-15; (c) Ti_3_C_2_T_x_-NaAsc-30; (d) Ti_3_C_2_T_x_-NaAsc-60. Figure S3: GCD curves. (a) Ti_3_C_2_T_x_-Fresh; (b) Ti_3_C_2_T_x_-NaAsc-15; (c) Ti_3_C_2_T_x_-NaAsc-30; (d) Ti_3_C_2_T_x_-NaAsc-60. Figure S4. Cyclic stability at 50 mV s^−1^. (a) Ti_3_C_2_T_x_-Fresh; (b) Ti_3_C_2_T_x_-NaAsc-15; (c) Ti_3_C_2_T_x_-NaAsc-30; (d) Ti_3_C_2_T_x_-NaAsc-60. The inserted indicates the comparison of CV curves between cycle 1 and cycle 10,000. Figure S5. Electrochemical measurements for Ti_3_C_2_T_x_-fresh and Ti_3_C_2_T_x_-H_2_O-30. (a) CV curves at 5 mV/s; (b) GCD curves at 1 A g^−1^. Table S1. Conductivity (S m-1) of various filtration membranes. Table S2. Specific capacitance (F g^−1^) of Ti_3_C_2_T_x_-fresh and Ti_3_C_2_T_x_-H_2_O-30.
