# Supplementary material for: Long-Term Storage of Ti3C2Tx Aqueous Dispersion with Stable Electrochemical Properties
Source: Materials (Basel). 2024 Nov 6;17(22):5414. doi: 10.3390/ma17225414 (PMC11595878; doi:10.3390/ma17225414)
Supplement: Supplementary file 1 [file materials-17-05414-s001.zip › materials-3230298-supplementary.pdf]

Supporting Information

Long-Term Storage of $Ti_3C_2T_x$ Aqueous Dispersion with Stable Electrochemical Properties

Ting Peng [1,2,†], Ruiqing Wu [1,†], Bohai Wang [1], Tomasz Liskiewicz [3,*] and Shengwei Shi [1,4,*]

[1] Hubei Key Laboratory of Plasma Chemistry and Advanced Materials, School of Materials Science and Engineering, Wuhan Institute of Technology, Wuhan 430205, China; pt@xjie.edu.cn (T.P.); 22305010031@stu.wit.edu.cn (R.W.); 22205010032@stu.wit.edu.cn (B.W.)
[2] School of Chemical and Environmental Engineering, Xinjiang Institute of Engineering, Urumqi 830002, China
[3] Faculty of Science and Engineering, Manchester Metropolitan University, Manchester M15 6BH, UK
[4] Key Laboratory of Optoelectronic Chemical Materials and Devices (Ministry of Education), Jianghan University, Wuhan 430056, China
* Correspondence: t.liskiewicz@mmu.ac.uk (T.L.), shisw@wit.edu.cn (S.S.)
† These authors contributed equally to this work.

Table S1. Conductivity (S m^{-1}) of various filtration membranes.

	0 d	15 d	30 d
**$Ti_3C_2T_x$-Fresh**	7.2×10^3	-	-
**$Ti_3C_2T_x$-H_2O**	7.2×10^3	$< 10^{-3}$	$< 10^{-3}$
**$Ti_3C_2T_x$-NaAsc**	7.2×10^3	6.7×10^3	6.3×10^3

Table S2. Specific capacitance (F g^{-1}) of $Ti_3C_2T_x$-fresh and $Ti_3C_2T_x$-H_2O-30

	5 mV/s	10 mV/s	20 mV/s	50 mV/s	100 mV/s	200 mV/s
**$Ti_3C_2T_x$-Fresh**	418.5	389.4	366.7	282.9	173.3	72.4
**$Ti_3C_2T_x$-H_2O-30**	226.9	210.5	189.1	150.5	113.6	72.3

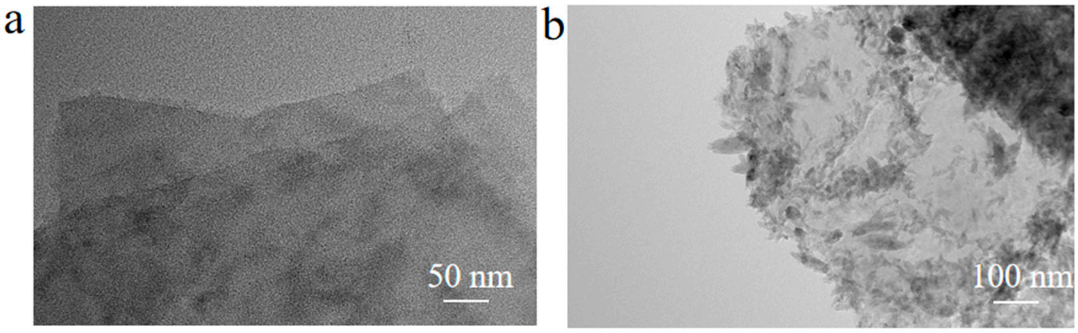

Figure S1. TEM images. (a) $Ti_3C_2T_x$-Fresh; (b) $Ti_3C_2T_x$-H_2O-30.

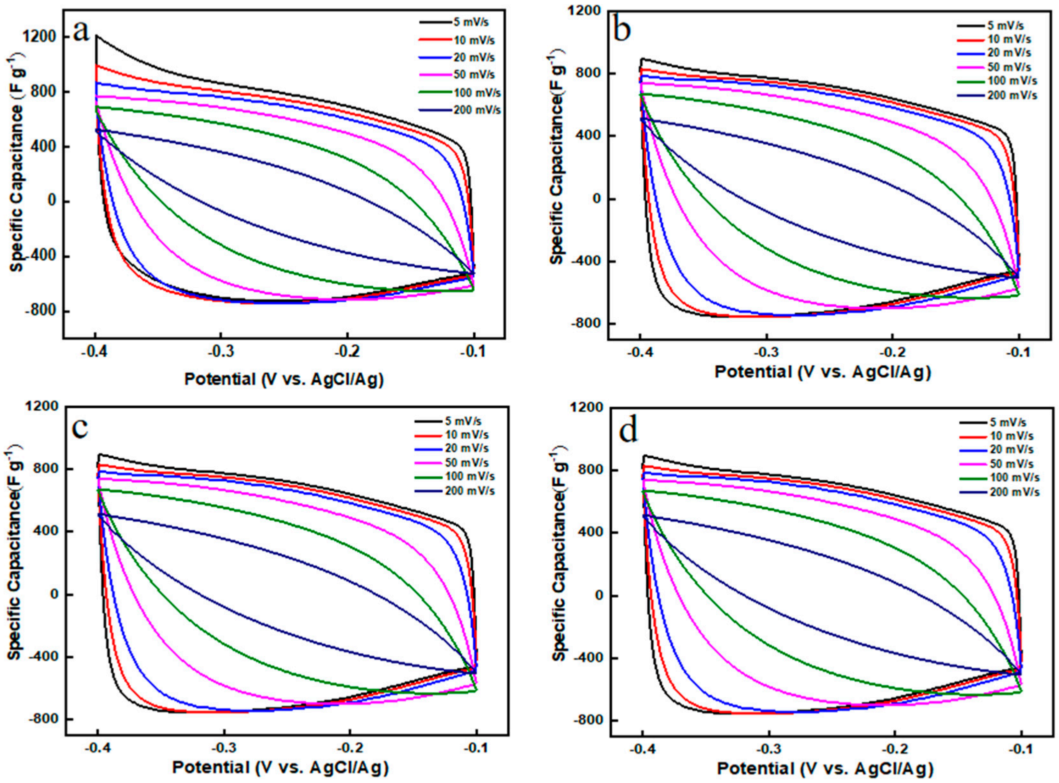

Figure S2. CV curves. (a) $Ti_3C_2T_x$-Fresh; (b) $Ti_3C_2T_x$-NaAsc-15; (c) $Ti_3C_2T_x$-NaAsc-30; (d) $Ti_3C_2T_x$-NaAsc-60.

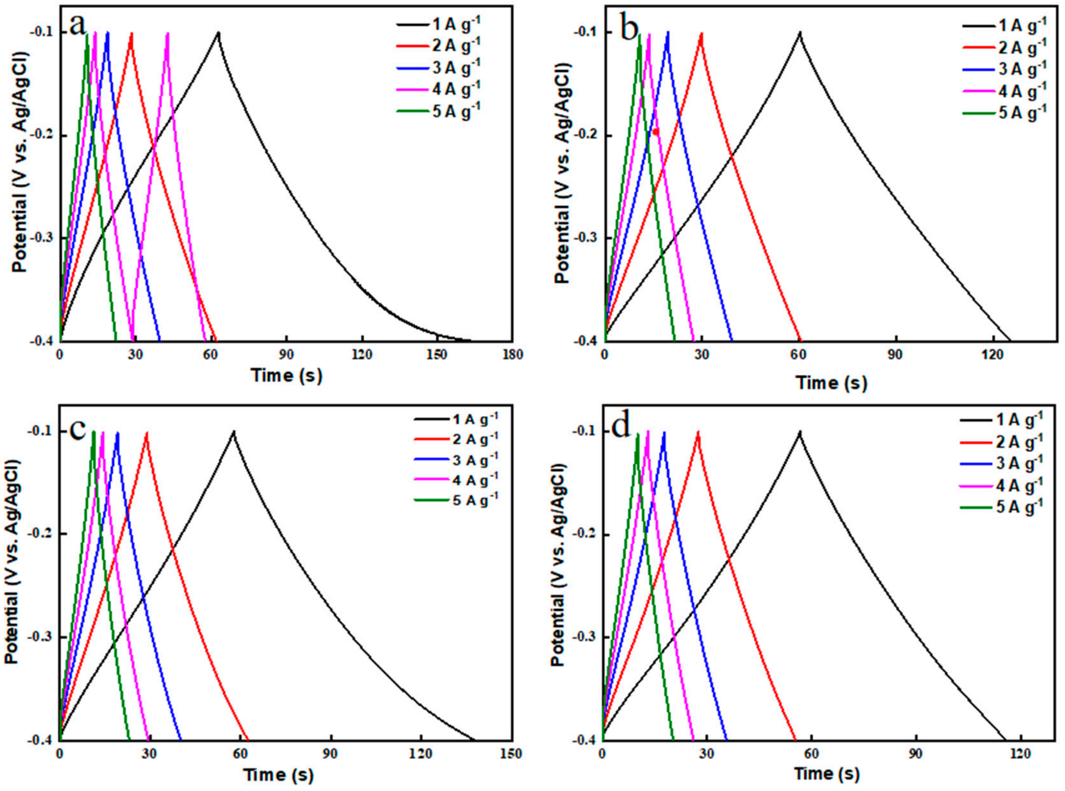

Figure S3. GCD curves. (a) $Ti_3C_2T_x$-Fresh; (b) $Ti_3C_2T_x$-NaAsc-15; (c) $Ti_3C_2T_x$-NaAsc-30; (d) $Ti_3C_2T_x$-NaAsc-60.

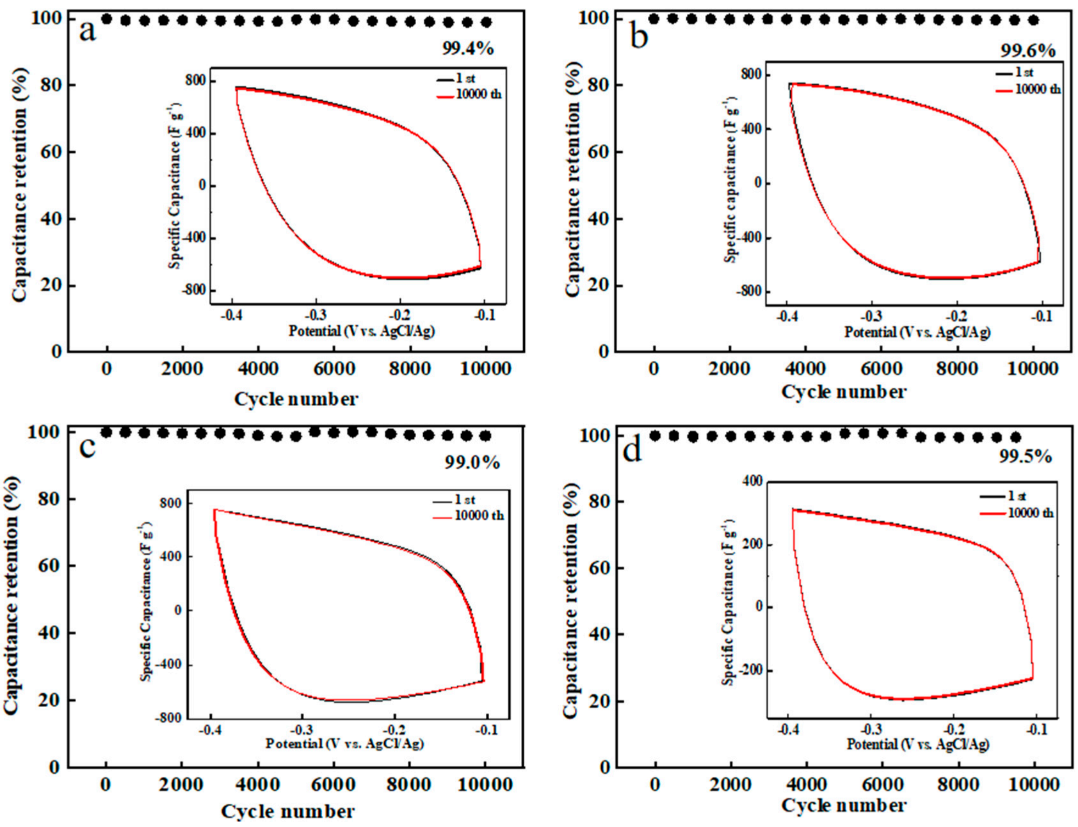

Figure S4. Cyclic stability at 50 mV s^{-1}. (a) $Ti_3C_2T_x$-Fresh; (b) $Ti_3C_2T_x$-NaAsc-15; (c) $Ti_3C_2T_x$-NaAsc-30; (d) $Ti_3C_2T_x$-NaAsc-60. The inserted indicates the comparison of CV curves between cycle 1 and cycle 10,000.

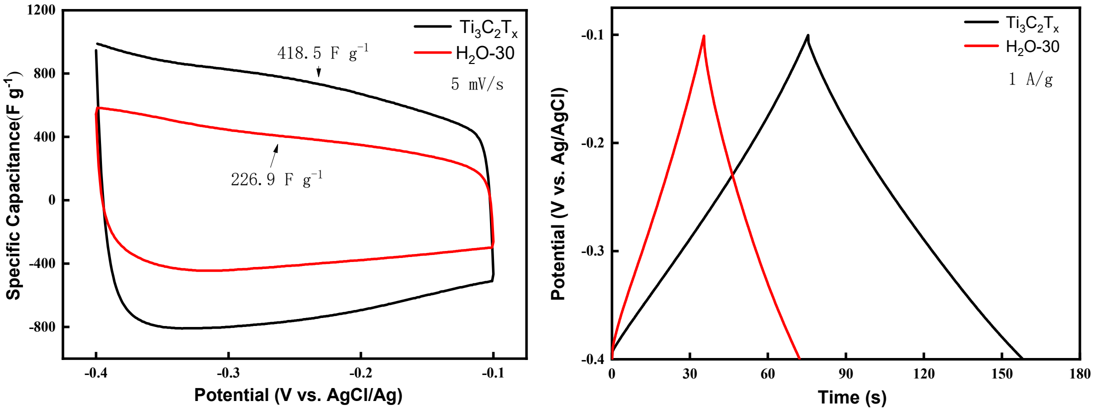

Figure S5. Electrochemical measurements for $Ti_3C_2T_x$-fresh and $Ti_3C_2T_x$-H_2O-30. (a) CV curves at 5 mV/s; (b) GCD curves at 1 A g^{-1}.
